# Biological Evaluation of the Copper/Low-density Polyethylene Nanocomposite Intrauterine Device

**DOI:** 10.1371/journal.pone.0074128

**Published:** 2013-09-18

**Authors:** Li-Xia Hu, Jing He, Li Hou, Hong Wang, Jun Li, Changsheng Xie, Zhuo Duan, Li-Kui Sun, Xin Wang, Changhong Zhu

**Affiliations:** 1 Family Planning Research Institute, Tongji Medical College, Huazhong University of Science and Technology, Wuhan, People’s Republic of China; 2 Central Hospital of Wuhan, Wuhan, P.R. China; 3 Shandong Quality Inspection Center for Medical Devices; Shandong Provincial Key Laboratory of Biological Evaluation of Medical Devices, Jinan, P.R. China; 4 Wuchang District Maternal and Child Health Hospital, Wuhan, P.R. China; 5 Department of Materials Science and Engineering, Huazhong University of Science and Technology, Wuhan, P.R. China; 6 Dayu Medical Devices Co., Ltd., Jingzhou, P.R. China; University of Illinois at Chicago, United States of America

## Abstract

Devices and materials intended for clinical applications as medical and implant devices should be evaluated to determine their biocompatibility in physiological systems. This article presents results from cytotoxicity assay of L929 mouse fibroblasts culture, tests for skin irritation, intracutaneous reactivity and sensitization, and material implantation tests for the novel copper/low-density polyethylene nanocomposite intrauterine device (nano-Cu/LDPE IUD) with potential for future clinical utilization. Cytotoxicity test *in vitro* was conducted to evaluate the change in morphology, growth and proliferation of cultured L929 mouse fibroblasts, which *in vivo* examination for skin irritation (n = 6) and intracutaneous reactivity (n = 6) were carried out to explore the irritant behavior in New Zealand White rabbits. Skin sensitization was implemented to evaluate the potential skin sensitizing in Hartley guinea pigs (n = 35). The materials were implanted into the spinal muscle of rabbits (n = 9). The cytotoxicity grade of the nano-Cu/LDPE IUD was 0–1, suggested that the composite was nontoxic or mildly cytotoxic; no irritation reaction and skin sensitization were identified in any animals of specific extracts prepared from the material under test; similarly to the control sides, the inflammatory reaction was observed in the rabbits living tissue of the implanted material in intramuscular implantation assay. They indicated that the novel composite intrauterine device presented potential for this type of application because they meet the requirements of the standard practices recommended for evaluating the biological reactivity. The nano-Cu/LDPE IUD has good biocompatibility, which is biologically safe for the clinical research as a novel contraceptive device.

## Introduction

The intrauterine device (IUD) is a safe, long-acting and effective method of contraception [1], [2]. Since the 1900s, many kinds of IUDs have been developed, and the copper-containing intrauterine device (Cu-IUD) is the most widely accepted [3]. However, use of the IUD has many side effects, such as pelvic pain, bleeding and spotting, etc. Many of these disadvantages occur within the first few months after Cu-IUD insertion [4], [5]. In order to decrease the side effects and increase contraceptive efficacy, nanotechnology was employed in the contraceptive field, and a new type of copper/low-density polyethylene nanocomposite IUD (nano-Cu/LDPE IUD) was invented by our research groups. The novel nano-Cu/LDPE IUD consists of high-quality copper nanoparticles and LDPE powders, synthesized by heating and evaporation methods [6]. Previous studies on this novel composite IUD, including material characteristics [7], [8], [9], [10], [11], corrosion behavior [6], [12], contraceptive efficacy and clinical performance [13], antifertility effectiveness and influence on the endometrial environment [14], [15], it is indicated that the nano-Cu/LDPE composites could greatly improve the performance of IUDs.

Evaluating biocompatibility of a material is an essential step toward the acceptance of the material in addition to testing of physical properties [16]. In recent years, the biological evaluation of biomaterials and medical devices has become more globally standardized, concurrently with the publication of the ISO 10993 standard for biomaterial and medical device testing. Biocompatibility assays involves testing either the material itself or an extract from it, or both, depending on the nature of the end-use application. *In vitro* cell culture studies are usually the first step of the evaluation of the biocompatibility. *In vivo* biological reactivity tests are designed to determine the biological response of animals to materials or medical devices with direct or indirect patient contact, or by the injection of specific extracts prepared from the material under test [17].

The aim of the present study is to evaluate the biocompatibility of this novel nano-Cu/LDPE IUD, such as cytotoxicity on L929 mouse fibroblasts by MTT assay *in vitro*, skin irritation, the guinea pig maximization test (GPMT) for skin sensitization, and muscle implantation *in vivo*, which may help us to examine the feasibility and safety of applying it as contraceptive device and to determine whether the material meets the biocompatibility standards for implantable medical products.

## Materials and Methods

### Samples

The nano-Cu/LDPE IUD was provided by the Department of Materials Science and Engineering of Huazhong University of Science and Technology. The total length of the transverse arms was 28 mm and total height of the longitudinal stem was 30 mm.

The TCu220C IUD was provided by Wuxi Medical Instrument Factory. The total length of the transverse arms was 30 mm; the total height of the longitudinal stem was 32 mm.

The cylindrical shape of Cu/LDPE nanocomposite and LDPE composite were provided by the Department of Materials Science and Engineering of Huazhong University of Science and Technology. The length of composites was 10 mm and diameter was 2 mm.

### Cytotoxicity Test: Test on Extracts and MTT Assay, *in vitro*

#### Preparation of the extracts

Preparation of reference materials for tests was based upon the International Organization for Standardization (ISO) standard ISO 10993-12 [18]. The complete culture medium was Dulbecco’s modified eagle medium (DMEM) (Amresco, Solon, OH, USA) supplemented with 10% (v/v) fetal bovine serum (FBS) (Amresco), with100 IU/ml penicillin, 100 μg/ml streptomycin and 2 mM/L glutamine (Shanghai Chemical Reagent Company, Shanghai, China). The nano-Cu/LDPE IUDs were put into the complete culture medium at the ratio of 1.25 cm^2^ sample in 1 ml solution. After the samples were soaked for 24 hours at 37°C incubator, the solution was disinfected and stored at 4°C.

Cytotoxicity test was carried out according to ISO 10993-5 [19]. Third-generation L929 fibroblasts (ATCC, CCL 1 NCTC clone 929 of mouse connective tissue) (Cell Bank of Chinese Academy of Sciences, Shanghai, China) were suspended at a concentration of 5×10^3^ cells/ml after detachment with 0.25% parenzyme and seeded in three 96-well cluster cell culture plates (each plate was inoculated with 60 wells). After 24 h, the complete culture medium was replaced with equal volumes (100 μl/well) of different experimental extracts (100%, 50%, 25%, 12.5% and 6.25%, v/v) of nano-Cu/LDPE and TCu220C IUDs, respectively. In negative control wells, fresh culture medium was added. Phenol (6.4 g/ml) was used as positive control. After 5 days of incubation, the shape of the fibroblasts in the experimental group and control group was examined with an inverted microscope (Olympus, Germany) and scanning electron microscopy (JEOL, Japan).

According to ISO 10993-5 [19], an MTT assay was used to evaluate the cytotoxicity of the composite. By measuring the optical density (OD) value of the formazan, the percentage of viable cells could be determined. The relative growth rate (RGR) was calculated by dividing the OD values of experimental wells by those of control wells and multiplying by 100. The cytotoxicity grade (CTG) of the specimen was obtained by the relationship between RGR and CTG. The sample meets the requirements of the test if all cell cultures exposed to the extracts show CTG equal to 0, 1 or 2.

### Experiments *in vivo*

Animal experimentations were conducted in compliance with all applicable provisions of the national laws, i.e., the Experiments on Animals Decree and the Experiments on Animals Act, and used the ARRIVE (Animal Research: Reporting of *In Vivo* Experiments) guidelines as a reference [20], [21], [22].

### Tests for Irritation and Intracutaneous Reactivity

The protocol was approved by the Committee on the Ethics of Animal Experiments of Shandong Quality Inspection Center for Medical Devices (Permit Number: IACUC-2010-043).

These test procedures were carried out according to ISO 10993-10 [23]. Adult healthy New Zealand White rabbits (Certificate No. 0009842) weighing 2.5–3.0 Kg were obtained from Lukang Pharmaceutical Co., Ltd., Shandong, China, which were individually housed and maintained under standard conditions (12-h light/dark cycle, 20±3°C, 50–60% relative humidity), with conventional laboratory diets and unrestricted supply of drinking water [24].

#### Skin irritation assay

Each of six young New Zealand White rabbits had their back hair clipped so as to expose a sufficient distance on both sides of the spine, which was divided into three groups of sites-the test sample group (nano-Cu/LDPE), the control sample group (TCu220C) and the blank control group (physiological saline solution). The test protocols were adopted for evaluating the extract liquids of nano-Cu/LDPE and TCu220C samples. The nano-Cu/LDPE and TCu220C IUDs were put into physiological saline solution at the ratio of 1.25 cm^2^ sample in 1 ml solution for 24 h at 37°C. After 24, 48 and 72 h of the application of control and test samples on the respective sites, the materials were removed and the sites were evaluated for erythema and edema according to the scoring system for skin reaction (Table 1) [23]. The primary irritation index (PII) for each animal is the sum of scores for erythema and edema at 24, 48 and 72 h after removal and divide by six. The cumulative irritation index is equal to the sum of the PIIs for each substance for all test animals divided by the number of test animals. The sample meets the requirements of the test if the cumulative irritation index is 0 to 0.4 [23].

**Table 1:** Scoring system for skin reaction [23].

Reaction	Irritation score
Erythema and eschar formation	
No erythema	0
Very slight erythema (barely perceptible)	1
Well-defined erythema	2
Moderate erythema	3
Severe erythema (beet-redness) to eschar formation preventing grading of erythema	4
Oedema formation	
No oedema	0
Very slight oedema (barely perceptible)	1
Well-defined oedema (edges of area well-defined by definite raising)	2
Moderate oedema (raised approximately 1 mm)	3
Severe oedema (raised more than 1 mm and extending beyond exposure area)	4
Maximal possible score for irritation	8

#### Animal intracutaneous reactivity test

Six healthy young adult New Zealand White rabbits were divided into two groups-the nano-Cu/LDPE group and the TCu220C group. Within a 24 h period before testing, rabbits exposed both sides of the spine. For each group of sides, one side was polar and non-polar extract and the other was polar and non-polar solvent control. The nano-Cu/LDPE and TCu220C IUDs were put into vegetable oil and physiological saline solution at the ratio of 1.25 cm^2^ sample in 1 ml solution for 24 h at 37°C. The vegetable oil and physiological saline solution were used as control substances. The extract of the nano-Cu/LDPE IUD obtained with physiological saline (0.2 ml) was injected intracutaneously on five sites on one side of each rabbit of nano-Cu/LDPE group. The extract obtained with vegetable oil (0.2 ml) was injected on five posterior sites on the same side of each rabbit. Similarly, 0.2 ml physiological saline and vegetable oil were injected on five sites of the contralateral side of each rabbit. For the TCu220C group, the procedures described preciously were repeated. The gross appearance at each injection site was observed immediately after injection and at 24, 48 and72 h after injection. The tissue reaction for erythema and oedema was graded according to the system given in Table 1 [23]. After the 72 h grading, all erythema grades plus oedema grades 24 h, 48 h and 72 h were totaled separately for each test sample or control for each individual animal. The score of a test sample or blank on each individual animal was calculated to divide each of the totals by 15 [3 scoring time points × 5 test or blank sample injection sites]. The overall mean score for each test sample and each corresponding blank was calculated to add the scores for the three animals and divided by three. The final test sample score could be obtained by subtracting the score of the blank from the test sample score. The requirements of the test are met if the final test sample score is 1, 0 or less [23].

### Skin Sensitization: Guinea Pig Maximization Test (GPMT)

The protocol was approved by the Committee on the Ethics of Animal Experiments of Shandong Quality Inspection Center for Medical Devices (Permit Number: IACUC-2010-042).

The test procedures were conducted in accordance with ISO 10993-10 [23].Thirty-five healthy young adult female White Hartley guinea pigs weighing 310–420 g (Certificate No. 0009464, Shanghai Shengwang experimental animal breeding Co., Ltd., Shanghai, China) were maintained under standard conditions (12-h light/dark cycle, 20±3°C, 30–70% relative humidity), supplied with guinea pig feed and unlimited drinking water [24]. The nano-Cu/LDPE IUDs were put into physiological saline and vegetable oil at the ratio of 1.25 cm^2^ sample in 1 ml solution for 24 hours at 37°C. For physiological saline vehicle, 10 guinea pigs (the experimental group) were for the sample extracts and 5 (the vehicle control group) for the blank extract liquid. For vegetable oil vehicle, 10 guinea pigs (the experimental group) were for the sample extracts and 5 (the vehicle control group) for the blank extract liquid. Five animals acted as a positive control group.

Induction phase: On day 0 (intradermal induction phase): Three pairs of 0.1 ml intradermal injection sites (A, B and C) of each animal were made simultaneously in the clipped interscapular region. Site A: A 1∶1 (v/v) mixture of Freund’s complete adjuvant (FCA)/physiological saline. Site B: Extracts of test sample. Site C: The extracts in a 1∶1 (v/v) mixture of (FCA)/physiological saline. Vehicle control groups received applications without test substance. Positive controls were treated with 1 g/L 1-chloro-2, 4-dinitrophenylhydrazine solution. On day 7 (topical induction phase): Approximately 24 h before the topical induction treatment the test areas were pretreated with 10% sodium dodecyl sulfate. For the topical induction, a patch with test extracts was applied to the clipped skin for 48 h. Control animals received the vehicle without the test extracts.

Challenge phase: The clipped skin of both test and control animals were challenged with the test extracts patches on day 21 for 24 h. About 24, 48 and 72 h after removal of the challenge patch, the skin reaction was observed and recorded according to the Magnusson and Kligman scale for each challenge site and at each time interval. If during the observation period none of the animals treated with the extract of the sample shows a greater grade than the animals treated with the blank, the sample meets the requirements of this assay [23].

### Material Implantation Test

The protocol was approved by the Committee on the Ethics of Animal Experiments of Shandong Quality Inspection Center for Medical Devices (Permit Number: IACUC-2010-046).

Implantation experiments were carried out according to ISO 10993-6 [25]. Nine healthy young adult New Zealand White rabbits (Certificate No. 0009842, Lukang, Shandong, China) weighing 2.5–3.0 Kg were divided into three groups of different implantation periods (1, 4 and 12 week), with three animals tested at each time period. Animals were anesthetized by intraperitoneal injection of sodium pentobarbital (30 mg/kg body weight). The Cu/LDPE nanocomposite with a length of 10 mm and diameter of 2 mm was implanted into the paravertebral muscle along one side of the spine at four points, at a 1.5 cm in depth and about 25 mm apart from each other. The contralateral side was implanted the control LDPE composites. Rabbits were sacrificed by CO_2_ asphyxiation sequentially at 1, 4, and 12 weeks after the implantation. Both sites of the spinal muscles adjacent to the materials were carefully excised from the surrounding tissue and made into paraffin section. The stained sections were assessed with light microscopy (Olympus, Japan).

### Statistics

All values were expressed as means ± standard (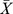
± S). Statistical analysis of the data was performed using one-way analysis of variance (ANOVA) and Student-Newman-Keuls test to determine differences in cytotoxicity based on specimen and incubation periods. A *p* value of <.05 was considered statistically significant.

## Results

### Evaluation for Cytotoxicity Test

**Figure 1:**
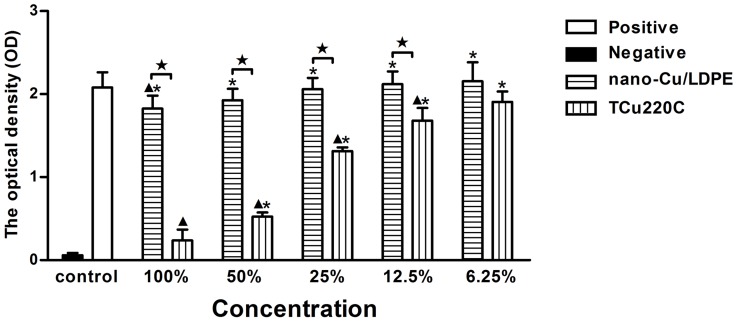
The OD values of the extracts of the nano-Cu/LDPE and TCu220C IUD for 1 d. *p<.05 compared with the positive group; ^▴^ *p*<.05 compared with the negative group; ^★^ *p*<.05 between two groups.

**Figure 2:**
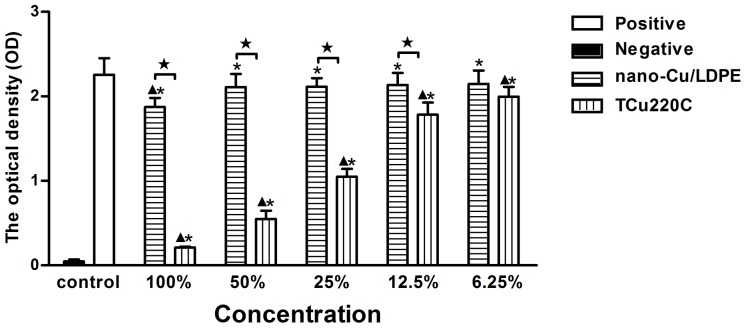
The OD values of the extracts of the nano-Cu/LDPE and TCu220C IUD for 3 d. *p<.05 compared with the positive group; ^▴^ *p*<.05 compared with the negative group; ^★^ *p*<.05 between two groups.

**Figure 3:**
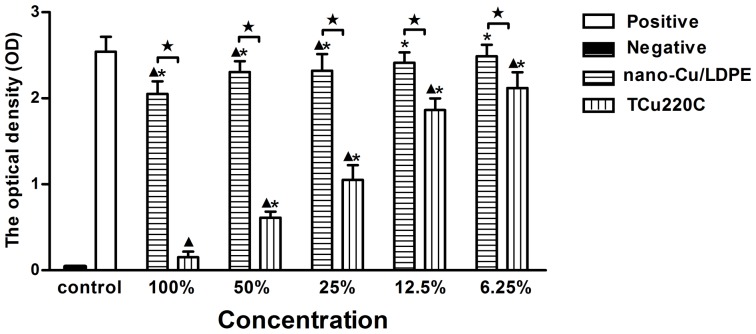
The OD values of the extracts of the nano-Cu/LDPE and TCu220C IUD for 5 d. *p<.05 compared with the positive group; ^▴^ *p*<.05 compared with the negative group; ^★^ *p*<.05 between two groups.

The optical density (OD) values indicate the percentage of viable cells. At 1 and 3 d period, there were no significant differences between the extracts of nano-Cu/LDPE IUD (50%, 25%, 12.5% and 6.25%, v/v) and negative control (*p*>.05). The OD values of the extracts of TCu220C IUD were significantly lower than nano-Cu/LDPE IUD and negative control except for 6.25% extracts (*p*<.05) (Figure 1 and 2). At 5 d period, there were no significant differences between the extracts of nano-Cu/LDPE IUD (12.5% and 6.25%) and negative control (*p*>.05). The OD values of all extracts of TCu220C IUD were significantly lower than nano-Cu/LDPE IUD and negative control (*p*<.05) (Figure 3). The RGR and CTG of extracts were shown in Table 2.

**Table 2:** Cytotoxicity assay determined as RGR and CTG of the L929 mouse fibroblasts.

Group	1 d		3 d		5 d	
	RGR	CTG	RGR	CTG	RGR	CTG
100% nano-Cu/LDPE	87.82	1	83.07	1	80.74	1
50% nano-Cu/LDPE	92.68	1	93.54	1	90.71	1
25% nano-Cu/LDPE	99.05	1	93.75	1	91.28	1
12.5% nano-Cu/LDPE	101.94	0	94.64	1	94.91	1
6.25% nano-Cu/LDPE	103.63	0	95.18	1	97.87	1
100% TCu220C	11.41	4	9.25	4	6.02	4
50% TCu220C	25.26	3	24.41	4	24.11	4
25% TCu220C	63.18	2	46.52	3	41.33	3
12.5% TCu220C	80.76	1	79.03	1	73.30	2
6.25% TCu220C	91.72	1	88.47	1	83.39	1
Positive control	2.79	4	2.03	4	1.68	4
Negative control	100	0	100	0	100	0

RGB: relative growth rate; CTG: cytotoxicity grade.

**Figure 4:**
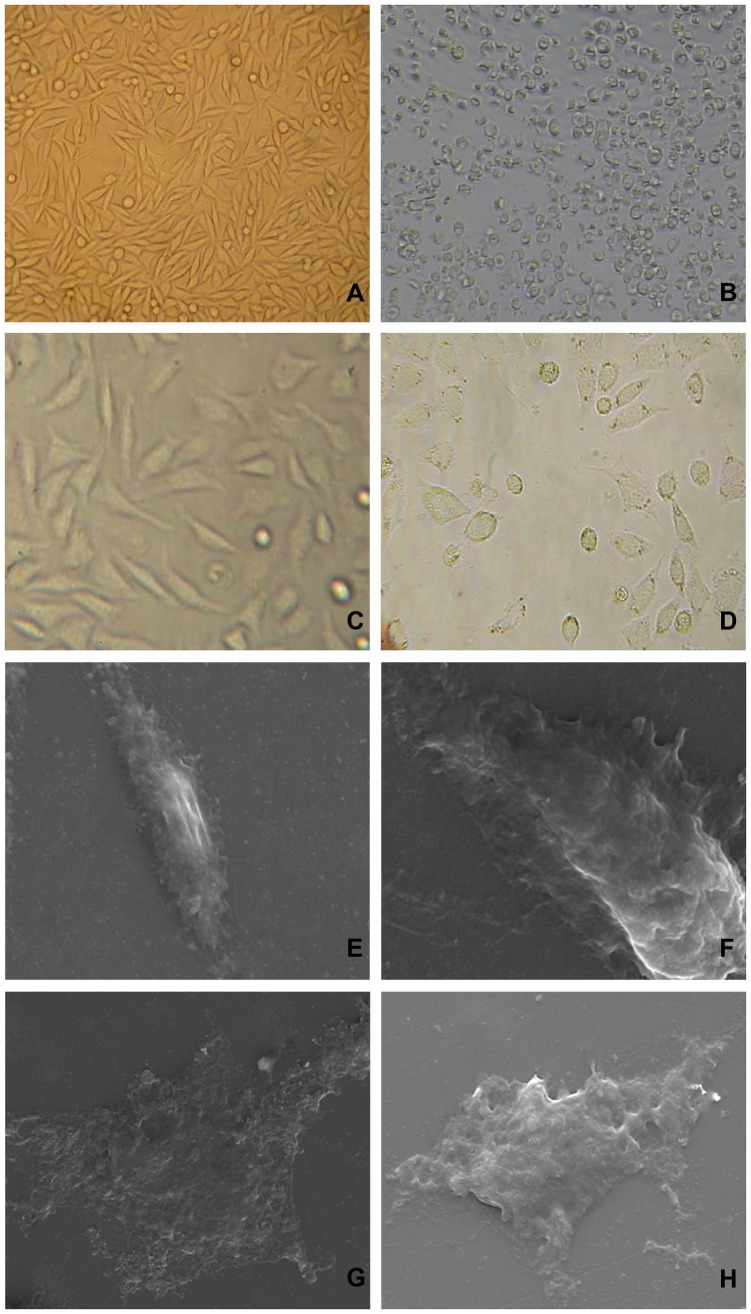
Morphologies of the cultured L929 mouse fibroblasts for 3 and 5 days. Morphologies with the light microscopy of the cultured L929 mouse fibroblasts for 3 days and extracts test. (A) negative control (×100): Fibroblasts were polygonal and cell division could be observed; (B) positive control (×200): Dying and dead fibroblasts were significantly smaller and floating. (C) nano-Cu/LDPE IUD-50% extracts (×400): Fibroblasts were polygonal and cell division could be observed. A very small amount of cells were round; (D) TCu220C IUD-50% extracts (×400): An amount of fibroblasts were dying and dead. Morphologies with scanning electron microscopy of the L929 mouse fibroblasts cultured with 50% extracts for 5 days. (E) nano-Cu/LDPE IUD-50% extracts (×3000): Fibroblasts membrane was integral; (F) nano-Cu/LDPE IUD-50% extracts (×6000): Membrane surface and the edge had long microvillus. (G) TCu220C IUD-50% extracts (×3000): Cells disintegrated and became larger. The cytoplasm was spillover; (H) TCu220C IUD-50% extracts (×4000): Fibroblasts were partial rupture.

The morphologies of the cultured fibroblasts with an inverted microscope for 3 days and scanning electron microscopy for 5 days were shown in Figures 4. The morphologies of the L929 fibroblasts shown the excellent cytocompatibility of the nano-Cu/LDPE IUD extracts.

### Tests for Irritation and Intracutaneous Reactivity

#### Skin irritation evaluation

The cumulative irritation index for the control, nano-Cu/LDPE and TCu220C IUDs presented a score of zero for 24, 48 and 72 h after removal of the test extracts.

#### Intracutaneous reactivity evaluation

The nano-Cu/LDPE IUD extracts as well as TCu220C IUD extracts displayed same reaction scores as control sites. All samples presented a final score of zero for 72 h after injection of the test extract for all sites assayed.

### Skin Sensitization Evaluation

The skin reactions except the positive group elicited no responses at the 24, 48 and 72 h readings after the challenge. For the positive group, all animals shown a grade 2–3 skin reaction at all reading time points after the challenge.

### Material Implanting Test

**Figure 5:**
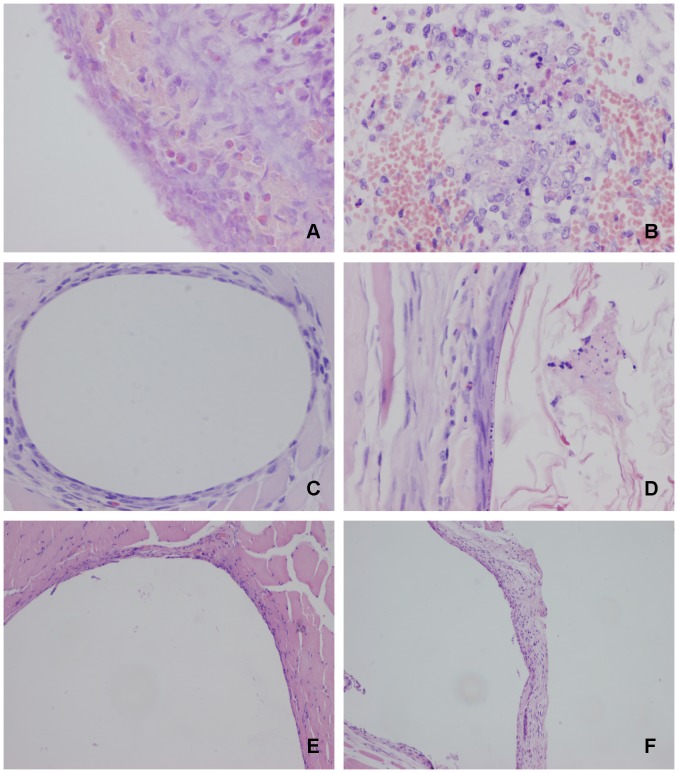
Light micrographs of muscle implantation histologic sections. Light micrographs of the nano-Cu/LDPE composite (B, D, F) which was implanted into the spinal muscle of adult rabbits compared with LDPE control material (A, C, E) for 1 (A, B), 4 (C, D) and 12 weeks (E, F). At 1 week, acute inflammatory reaction was the main characteristic (A, B) (×400). A lot of heterophil granulocytes and a small amount of lymphocytes infiltration were seen around the nano-Cu/LDPE implant, but without formation of complete capsule (B). Similar inflammatory reaction was observed on the LDPE composite side (A). After 4 weeks of implantation, the number of heterophil granulocytes decreased sharply, and lymphocytes were seldom. Fibrous capsules formed on the test and control materials surface were clearly observed (C, D) (×200). At 12 weeks, the inflammatory reaction of the two group sites had almost disappeared. Furthermore, the surrounding fibrous capsule had become more thinning and stable (E, F) (×100).

For all of the rabbits, the insertion hole could still be found on the muscle fascia for nano-Cu/LEPE and LDPE materials after 1, 4, and 12 weeks. After 1 week of implantation, the implants were located in a muscular pocket that was clearly separated from the surrounding tissue. At 4 weeks, fibrous tissue formation encircled the implanted materials. At 12 weeks, the implants adhered tightly to the surrounding tissue. Light micrographs of sections after 1, 4, and 12 weeks of implantation were shown in Figure 5. Preliminary observations indicated that the two types of materials had equal implantation behavior and reaction.

## Discussion

The biological behavior of the novel nano-Cu/LDPE IUD was investigated to evaluate the response of cell cultures and animals to the material by direct or indirect patient contact, or by injection of their specific extract liquids, by using standard practices, which recommend generic biological tests for materials, medical devices, and implants for human application [17].

The ISO-10993-5 guidelines for “Biological Evaluation of Medical Devices” allow for the use of MTT assay and extract assay in cytotoxicity determinations [19]. The results of the present study indicated that the nano-Cu/LDPE IUDs were nontoxic or mildly cytotoxic at all incubation periods and concentrations, which meet the requirements of the cytotoxicity test to ensure the contraceptive efficacy. A significant decrease in cell viability was found for TCu220C IUD at all incubation periods except for 6.25% extracts at 1 d period. Contrary to these findings, in the present study, the current TCu220C IUD exhibited serious cytotoxic effect than the novel composite IUD. The contraceptive effect of Cu-IUD is mainly dependent on the copper ions released by the corrosion of copper [26], [27], [28], [29], [30], and the larger the release rate of cupric ion, the better the contraceptive effect. According to the literatures [31], there is an extremely high corrosive rate during the first few days known as cupric ion “burst release”, when Cu-IUD is immersed into the simulated uterine fluid. It is suggested that the higher concentration of Cu^2+^ in the initial period and the stronger cytotoxicity with the prolonged duration may be the main factors that induce adverse effects such as heavy bleeding and pelvic pain immediately following IUD insertion [32], [33], [34]. Previous *in vitro* research demonstrated that Cu^2+^ release rate from the Cu/LDPE nanocomposites can be modulated by changing the copper nanoparticle mass fraction and that all the samples display near zero-order release after a month of incubation [6]. With the same content of copper, the nano-Cu/LDPE IUD releases more copper ions than the conventional Cu-IUD in the same liquid environment [14]. The antifertility effect of the Cu/LDPE nanocomposite is as excellent as that of the Cu-IUD material [15]. Therefore, the cytotoxicity caused by the nano-Cu/LDPE IUD is much less than that caused by TCu220C IUD.

The skin irritation assay and intracutaneous reactivity test evaluate the irritation potential of a biomaterial by contact with skin and intradermal injection of rabbits [23]. The results of the present study indicated that the nano-Cu/LDPE IUD and TCu220C IUD exhibit a nonirritant behavior and no inflammatory response to edema or erythema formation. The GPMT is a reliable method developed by Magnusson and Kligman [35] that employs Freund’s Complete Adjuvant. The data presented in this study clearly demonstrated that nano-Cu/LDPE IUD had not lead to skin sensitization in the GPMT. The GPMT was designed largely for hazard identification and requires further evaluation, that is, quantitative induction and elicitation data, for proper risk assessment in the clinic [36].

The experimental materials were regarded to be biocompatible if the intensity of connective reaction decreased over time. The low-density polyethylene (LDPE) possesses excellent biocompatibility with human body and usually used as implantable material. Consequently, to be considered biocompatible, at 90th days the connective tissue surrounding the implant must show a thin fibrous capsule formation surrounding the tube as well as an absence of inflammatory reaction and/or macrophages/giant cells. On the other hand, the material is considered to be non biocompatible when a persistent inflammatory reaction occurs related to macrophages and giant cells with a thick fibrous capsule development even at 90 days after implantation [25], [37].

In conclusion, this partial study on the biological reactivity of nano-Cu/LDPE IUD proposed suggests that the material presents potential for future applications as medical devices and implants according to the requirements of the standard practices recommended for evaluating the biocompatibility response. The existence of a correlation between *in vivo* biological reactivity tests results and those for tissue culture was observed. Although these assays are necessary, extrapolating this to the clinical situation in humans remains far from obvious. Additional studies involving acute systemic and subchronic toxicity, reproductive toxicity and genotoxicity tests to estimate the biological reaction of the composite are already in progress in order to assess the final suitability of the candidate medical device intended for end-use human applications.
